# Phlegmasia Cerulea Dolens: A Life-Threatening Manifestation of Deep Vein Thrombosis

**DOI:** 10.7759/cureus.8587

**Published:** 2020-06-12

**Authors:** Chung-ting J Kou, Caitlin Batzlaff, Matthew L Bezzant, Tyson Sjulin

**Affiliations:** 1 Internal Medicine, Brooke Army Medical Center, Fort Sam Houston, USA; 2 Pulmonary and Critical Care, Brooke Army Medical Center, Fort Sam Houston, USA

**Keywords:** venous thrombosis, deep venous thrombosis, phlegmasia cerulea dolens, endovascular catheter-directed thrombolysis, gangrene, thrombectomy

## Abstract

Deep vein thrombosis is a common condition encounter by hospitalists and managed by either oral or intravenous anti-coagulation. Although uncommon, phlegmasia cerulea dolens (PCD) is a life-threatening manifestation of acute deep vein thrombosis requiring early recognition and aggressive intervention to preserve life and limb. PCD is characterized by marked swelling of the lower extremities with pain and cyanosis, which often leads to gangrene and amputation. We present the case of a patient who developed PCD of her left lower extremity who was successfully treated with an EkoSonic™ endovascular catheter (Boston Scientific, Marlborough, MA, USA), which accelerates lytic dispersion of the thrombolytic drug through ultrasound technology.

## Introduction

Venous thromboembolism (VTE) is the third leading vascular emergency affecting approximately 300,000 to 600,000 Americans annually with significant morbidity and mortality [1]. Phlegmasia cerulea dolens (PCD) is a rare and extreme manifestation of deep vein thrombosis (DVT), which can result in gangrene, loss of limb, and ultimately death [2]. Venous gangrene develops due to obstructed arterial flow as a result of extreme venous hypertension [3]. The pathogenesis of PCD is related to increased hypercoagulability, stasis, and/or vascular wall injury, with malignancy as the most common risk factor [4,5]. It occurs more commonly in the fifth and sixth decades of life, with preferential involvement of the left leg [6]. The left lower extremity (LLE) is more commonly associated with PCD due to compression of the left common iliac vein by the right common iliac artery. [6] Currently, there is no consensus on a superior therapeutic intervention with endoscopic modalities including catheter-directed thrombolysis (CDT) and pharmacomechanical thrombolysis (PMT) to surgical interventions such as surgical thrombectomy. We report a case of PCD successfully treated with an EkoSonic™ (EKOS, Boston Scientific, Marlborough, MA, USA) endovascular catheter, which generates an acoustic field through ultrasound to enhance clot dissolution followed by stent placement and angioplasty.

## Case presentation

**Figure 1:**
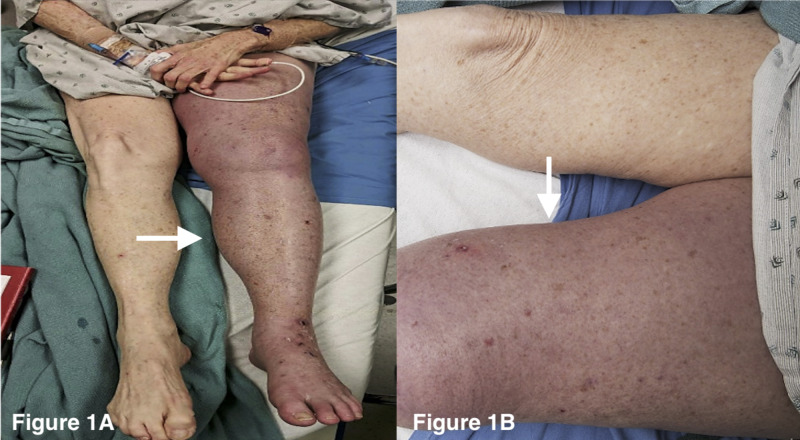
Patient's Lower Extremity at Presentation Physical examination of the left lower extremity demonstrating purplish discoloration, edema, warmth, tenderness to palpation with diminished dorsalis pedal and popliteal pulses. (A) Purplish discoloration and edema in the entire left lower extremity compared to the right lower extremity. (B) Close-up of the affected left quadriceps region highlighting the swelling and skin discoloration compared with the unaffected right quadriceps region.

A 66-year-old female presented to the emergency department with three days of acute LLE swelling, pain, cyanosis, inability to bear weight, dyspnea, and hypoxemia (Figure 1). She denied recent travel, hormone replacement, or a history of DVT/pulmonary embolism (PE). Her medical history was notable for systemic lupus erythematosus, CREST (calcinosis, Raynaud's phenomenon, esophageal dysmotility, sclerodactyly, and telangiectasia) syndrome, Sjogren’s syndrome, and local invasive anal squamous cell carcinoma. Doppler ultrasound revealed LLE thrombus extending from the greater saphenous vein to the popliteal vein. She was admitted to the intensive care unit (ICU) and started on an unfractionated heparin drip.

**Figure 2:**
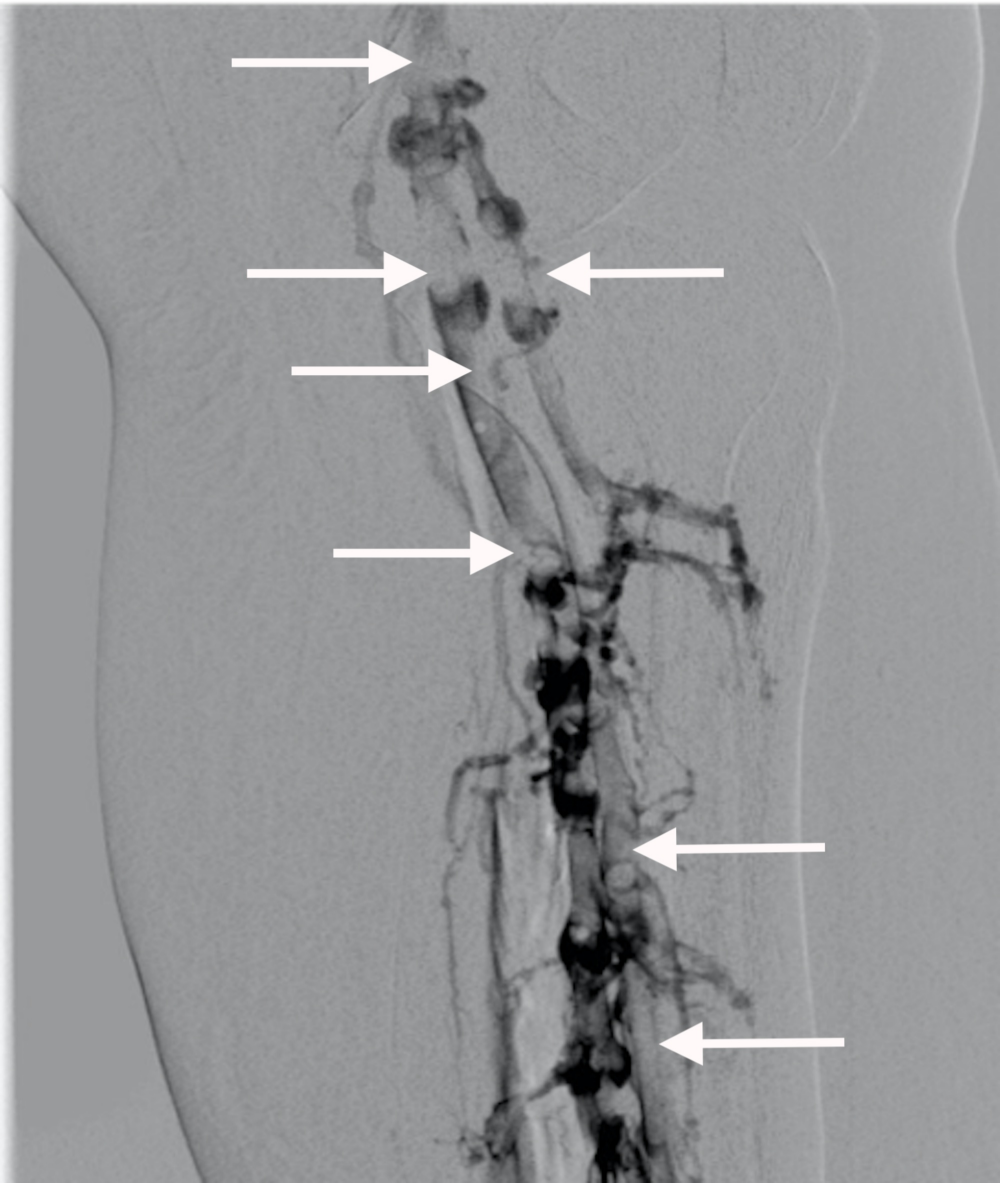
Left Lower Extremity Venogram Pre-Intervention Prior to catheter-directed thrombolysis, the left lower extremity venogram demonstrates extensive clotting of the tibial, popliteal, and femoral venous system. Clotting is demonstrated by the filling defects in the areas highlighted by the white arrows.

**Figure 3:**
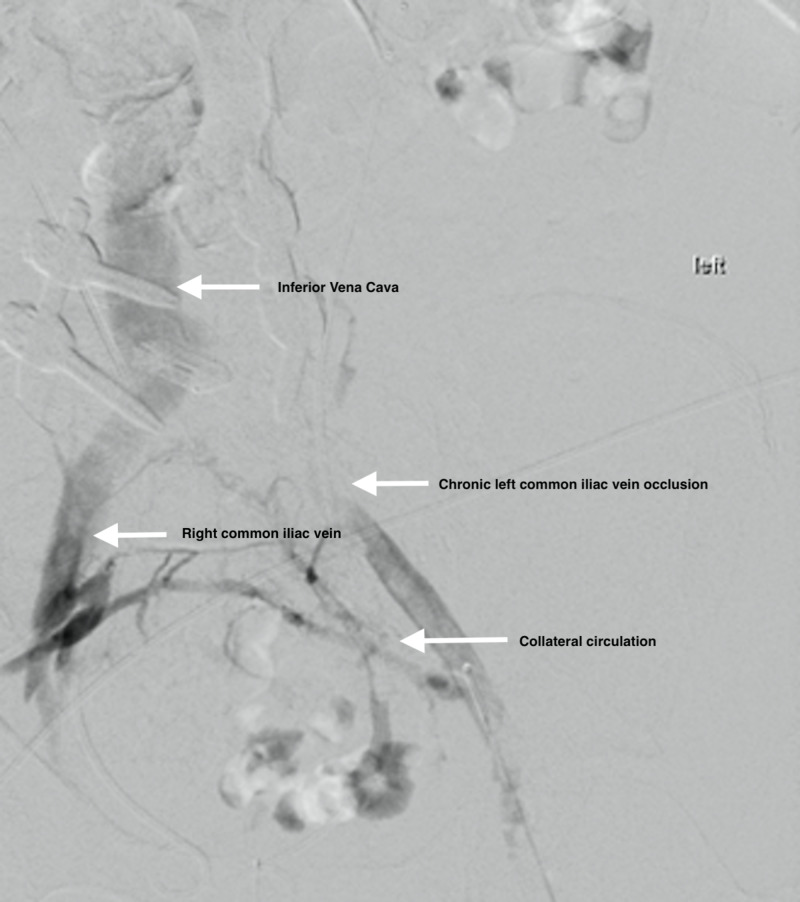
Chronic Left Iliac Occlusion Pre-Intervention Chronic occlusion of the left common iliac vein with venous drainage through the right collateral venous system into the right common iliac vein and into the inferior vena cava vein.

**Figure 4:**
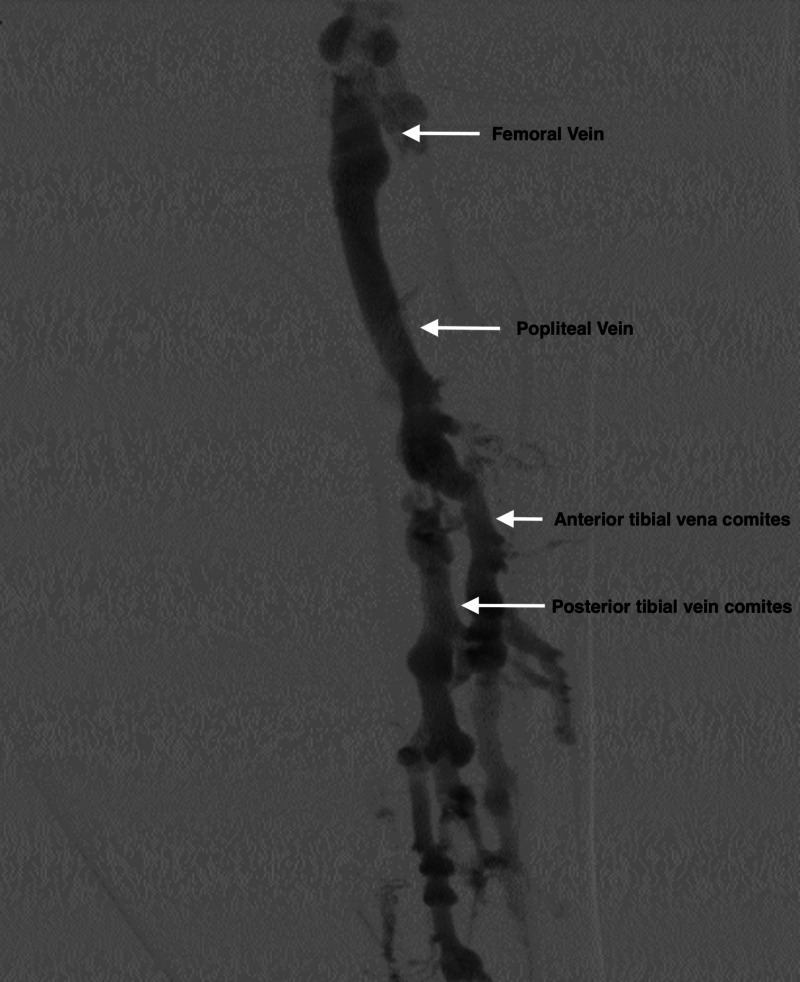
Left Lower Extremity Venogram Post-Intervention Venogram at 24 hours post-placement of an EKOS endovascular catheter in the left external iliac vein demonstrating overall clot burden in the tibial, popliteal and femoral venous system. EKOS endovascular catheters use ultrasound pressure with acoustic streaming and a thrombolytic agent to dissolve the thrombus. EKOS, EkoSonic

**Figure 5:**
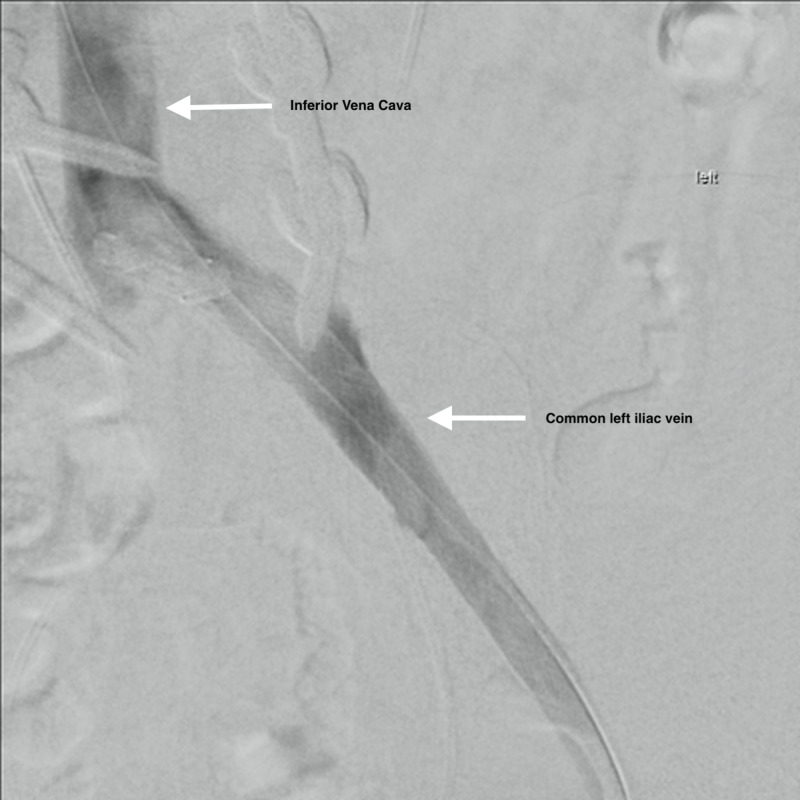
Left Chronic Iliac Occlusion Post-Intervention Left chronic common iliac occlusion demonstrating venous flow status post-placement of 20 x 80 mm Boston Scientific Wallstent (endoprosthesis stent) followed by balloon angioplasty with a 12 x 40 mm Atlas dilatation catheter. Further angioplasty of the chronic occlusion was achieved with a high pressure 8 x 40 mm Boston Scientific Mustang balloon dilatation catheter.

The vascular surgery team was consulted for surgical intervention. After further review of her case, the vascular surgery team elected for an endovascular approach with CDT of LLE. Initial venogram demonstrated extensive clotting of the tibial, popliteal, and femoral venous systems extending into the iliac system (Figures 2, 3). From a tibial approach, a guidewire was advanced up to the common iliac, but a chronic occlusion of the left common iliac vein was found, which could not be crossed. A venous collateral circulation system in communication with the right iliac venous system was identified. In order to decrease the overall clot burden, two 50-cm EKOS endovascular catheters were placed in the left external iliac vein and collateral system. Repeat venogram at 24 hours post-CDT showed significant improvement of the overall clot burden with almost complete resolution of the clot in the LLE; however, the chronic occlusion remained (Figure 4). A 20 x 80 mm Boston Scientific Wallstent™ (endoprosthesis stent) was advanced across the total chronic total occlusion and deployed. Post-operative venogram demonstrated significant improvement in flow in the system, with all flow going through the iliac system, and no flow further through the previously seen collaterals. Intravascular ultrasound was advanced through the area, which showed severe compression of the stent. A 12x 40 mm Atlas® dilatation catheter (Bard Peripheral Vascular Inc., Tempe, AZ, USA) was used for angioplasty. Repeat intravascular ultrasound showed persistent compression of the area. A 8 x 40 mm Boston Scientific Mustang™ dilatation catheter (high-pressure balloon) was used to re-attempt angioplasty; however, compression was still noted (Figure 5). Regardless, outflow improved throughout the left common iliac system, and no further interventions were performed. The patient returned to the ICU and transitioned to direct oral anticoagulation without any further complications. She was subsequently transferred to a medicine ward for further care.

 

 

## Discussion

PCD is a vascular emergency that requires early recognition and prompt coordination with either interventional radiology or vascular surgery to prevent irreversible tissue damage, amputation, and death. However, if there are contraindications to thrombolytic therapy or if clinical signs of compartment syndrome are present, a vascular surgery consultation is more appropriate. The pathophysiology of PCD involves complete obstruction of both superficial and deep venous return, resulting in increased interstitial tissue pressure, arrest of capillary flow, tissue ischemia, and, ultimately, gangrene [7,8]. Risk factors include malignancy, heparin-induced thrombocytopenia, femoral vein catheterization, anti-phospholipid syndrome, and pregnancy [4-5,9]. The most probable etiology of our patient's PCD is likely due to her malignancy history, as malignancy has been reported in approximately 20% to 40% of patients with PCD [5]. Due to the rarity of PCD, the annual incidence rate of the disease remains unknown. Furthermore, an extensive PubMed search reported amputation rates among survivors ranging from 12% to 50% and a reported mortality rate of 20% to 41% in various reported case series and case reports [6,8].

The diagnosis of PCD is a clinical diagnosis based on manifestations of three cardinal signs massive swelling, pain out of proportion, and discoloration or skin molting [5]. This is followed by cyanosis and sensory and/or motor strength of the extremity, with increasing impairment of the arterial circulation resulting in tissue ischemia and gangrene of the affected extremity [6]. Venous ultrasound remains the first diagnostic modality for DVT [6,10]. Accuracy studies comparing venography and duplex studies have demonstrated that venous ultrasound has a mean sensitivity and specificity of 97% and 94%, respectively, with a mean positive and negative predictive value of 96% and 98% for symptomatic, proximal DVT, respectively [11]. However, PCD can involve thrombosis of the iliac veins, which may not be visualized on venous ultrasound due to body habitus, depth, overlying bowel gas, and incompressibility of the retroperitoneal veins [11]. Computed tomography (CT) venography or magnetic resonance (MR) venography can be used as adjunctive imaging modalities to better characterize the extent of the proximal thrombus within the inferior vena cava and pelvic veins [11]. Unfortunately, no formal criteria exist to identify and risk-stratify the severity of PCD. Ultimately, the diagnosis of PCD is based on physical examination findings and radiographic evidence of DVT either on ultrasound and/or CT/MR venography.

The goal of PCD therapy is aggressive reduction in thrombus load and prevention of further thrombus propagation. Initial treatment of PCD is achieved with absolute bed rest, elevation of the affected extremity, fluid resuscitation, and administration of intravenous heparin [12,13]. The relative value of specific therapeutic modalities remains uncertain [12]. The extent of ischemia dictates whether an endovascular approach or a surgical approach is the most appropriate. The clinical severity of ischemia can be categorized on the basis of acute limb ischemia index (Table 1) developed by Rutherford et al., which is based on the level of sensory loss, muscle weakness, and the presence of lack of arterial as well as venous Doppler signal [14].

**Table 1:** Rutherford Acute Ischemia Index Source: Adapted from Rutherford [14].

Clinical Findings				Doppler Signal		Skin Examination
Rutherford Class	Prognosis	Sensory Impairment	Motor Impairment	Arterial	Venous	
Class I: Viable	Threatened	None	None	Audible	Audible	Normal capillary return
Class IIa: Threatened Marginally	Salvageable with prompt therapy	Minimal loss in toes or none	None	Often inaudible	Audible	Decreased capillary return
Class IIb: Threatened Immediately	Salvageable if treated immediately	Involves forefoot ± pain at rest	Mild to moderate	Usually inaudible	Audible	Pallor
Class III: Irreversible	Irreversible tissue and nerve damage	Anesthetic	Paralysis and rigor	Inaudible	Inaudible	No capillary return, molted skin

Our patient's presentation fell under class IIa of the acute ischemic index, and thus an endovascular approach was selected [14]. Limb-sparing endovascular revascularization modalities include CDT and PMT. In CDT, a catheter is placed endovascularly with delivery of a thrombolytic agent to the occluding thrombus. Absolute contraindications to CDT include active internal bleeding, previous stroke, neurosurgical intervention, and intracranial hemorrhage within three months [15]. In PMT, the catheter delivers the thrombolytic drug with concomitant thrombus aspiration or maceration [13,16]. Additionally, PMT catheters are useful in patients with contraindications to lytic therapy as the device can be used without a thrombolytic agent [15]. Potential disadvantages of catheter-guided therapy include prolonged thrombolytic infusion requiring ICU level care, possible displacement of the thrombus from mechanical manipulation resulting in PE, and venous valve damage [14,15]. The shortcomings of CDT have been documented by Lin et al. in a retrospective study of 93 patients with symptomatic lower extremity DVT undergoing catheter-directed interventions. Compared with the CDT group, the PMT group had lower rates of ICU stays, shorter hospitalizations, and fewer packed red blood transfusion requirements [17]. CDT requires a prolonged ICU course secondary to long-lytic infusion times, whereas ultrasound-accelerated thrombolysis (UAT) uses less lytic agent and has a shorter infusion time. Given this, a UAT approach with an EKOS catheter was selected. The principle mechanism of EKOS is similar to PMT. However, EKOS uses high-frequency low-power microsonic energy to temporarily loosen and separate fibrin, thus augmenting the delivery of a lytic agent to the thrombus [18]. As a result, our patient required only a 24-hour ICU stay. If there is profound ischemia at presentation, as categorized by class IIB of the acute ischemia index, then a surgical approach may be more appropriate [6]. Venous thrombectomy is a surgical modality that provides rapid relief of venous and compartmental hypertension, prevents further thrombus propagation, and avoids post-thrombotic sequela [13]. However, drawbacks include recurrent thrombosis, long-term compression stocking use, and general anesthesia [19]. If gangrene has occurred, amputation of the affected limb is indicated [6,13]. As the literature regarding PCD continues to evolve, more data will be available to establish a standard endovascular and surgical therapeutic modality based on the ischemic severity of PCD at presentation.

## Conclusions

Our case demonstrates an extreme clinical presentation of VTE requiring emergent CDT and angioplasty with stenting in order to preserve tissue. Early recognition of PCD and rapid coordination with vascular surgery or interventional radiology team is critical in the preservation of limb and life, as occurred in our case. Due to the rarity of PCD, additional data are necessary to evaluate the clinical efficacy of UAT compared with other endovascular modalities for PCD cases with an ischemic index of class IIA and less, as demonstrated in our case. For now, selecting the optimum therapeutic intervention is largely guided by the ischemic severity on presentation, the type of endovascular catheter that is available, and the comfort level of the interventionist/surgical specialist performing the intervention.
